# Neural mechanisms of pain susceptibility and resilience: Insights from a socially transferred allodynia mouse model

**DOI:** 10.1016/j.jatmed.2025.09.002

**Published:** 2025-09-26

**Authors:** Yi Han, Lin Ai, Xin Yang, Hongxing Zhang

**Affiliations:** a Department of Bioengineering, University of Pittsburgh, Pittsburgh, PA 15260, USA; b Department of Anesthesiology, Daping Hospital, Army Medical University, Chongqing 400042, China; c Jiangsu Province Key Laboratory of Anesthesiology, Xuzhou Medical University, Xuzhou 221004, China; d Jiangsu Province Key Laboratory of Anesthesia and Analgesia Application Technology, Xuzhou Medical University, Xuzhou 221004, China; e NMPA Key Laboratory for Research and Evaluation of Narcotic and Psychotropic Drugs, Xuzhou Medical University, Xuzhou 221004, China

Pain is a universal yet highly subjective experience, influenced by a complex interplay of biological, psychological, and social factors.^1^ Inter-individual differences in pain sensitivity have long been observed in humans, with some individuals reporting severe pain while others exhibit resilience under similar conditions.^2^ For instance, rare outlier individuals among patients with inherited erythromelalgia report experiencing less pain, whereas the majority suffer from intolerable, intense pain.^3^ However, despite the ubiquity of these differences in humans, they have been difficult to replicate in laboratory animals. Traditional pain models, which often rely on tissue damage or inflammation,^4^, ^5^ fail to capture the nuanced variability in pain responses observed in human populations. This has limited our understanding of the neural mechanisms underlying pain susceptibility and resilience.

Our recent studies by Ai et al. ^6^ and Han et al. ^7^ address this issue by employing a well-established socially transferred allodynia (STA) model.^8^ In this model, only a subpopulations of mice exhibit pain-like behaviors after a brief social contact with a cage mate experiencing inflammatory pain.^7^ This approach not only recapitulates the social transfer of pain but also allows for the identification of distinct subpopulations of mice that are either susceptible (BY-S) or resilient (BY-R) to STA. The researchers established two independent criteria for separation: a paw withdrawal threshold (PWT) of 0.41 g and a PWT ratio of 75 % (the ratio of PWTs after versus before the brief social contact).^7^ BY mice with PWTs ≤ 0.41 g are defined as susceptible, while those with PWTs > 0.41 g are classified as resilient. Similarly, BY mice with a PWTs ratio lower than 75 % are categorized as susceptible, and those with a ratio greater than 75 % are deemed resilient (Fig. 1). Notably, over 90 % of BY-S and BY-R animals identified using these two different cutoffs overlapped. By establishing this framework, these studies provide a practical strategy for distinguishing susceptible and resilient subpopulations in mice exposed to the same brief social contact with a cage mate experiencing inflammatory pain. This model serves as a valuable tool for studying inter-individual differences in pain sensitivity.^9^, ^10^

**Fig. 1:**
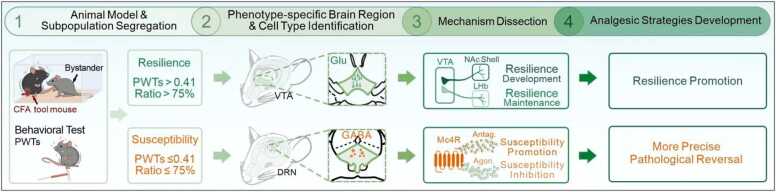
Using a mouse model of socially transferred allodynia, Han and Ai et al. established two behavioral criteria to distinguish susceptible and resilient subpopulations, identifying two nuclei that respectively regulate related behavioral phenotypes. The ventral tegmental area glutamatergic neurons were found to control the development and maintenance of resilience to socially transferred allodynia through their projections to the nucleus accumbens shell and lateral habenula, respectively. DRN GABA-ergic neurons and their enriched Mc4R specifically mediate the susceptibility to socially transferred allodynia. These findings not only provide a novel therapeutic strategy for pain management by promoting resilience mechanisms, but also enable more precise interventions by targeting susceptible mechanisms. CFA, complete Freund’s adjuvant; PWTs, paw withdrawal thresholds; VTA, ventral tegmental area; DRN, dorsal raphe nucleus; NAc, nucleus accumbens; MC4R, melanocortin 4 receptor; Antag, antagonist; Agon, agonist.

The study by Ai et al. focuses on the role of dorsal raphe nucleus (DRN) GABAergic neurons in pain susceptibility (Fig. 1).^6^ Using a combination of chemogenetic, molecular, pharmacological, and electrophysiological approaches, the researchers demonstrated that DRN GABAergic neurons are selectively activated in BY-S mice.^6^ Chemogenetic activation of these neurons promoted STA susceptibility, while their inhibition prevented the development of STA and reversed established STA behaviors. Furthermore, based on their previous findings,^11^ the study identified the melanocortin 4 receptor (MC4R) as a molecular target within DRN GABAergic neurons that regulates pain susceptibility. Pharmacological activation of MC4R reduced pain susceptibility, whereas its antagonism increased it.

In contrast, Han et al. identify ventral tegmental area (VTA) glutamatergic neurons as key cellular substrates for pain resilience (Fig. 1).^7^ The researchers demonstrated that VTA glutamatergic neurons are selectively activated in BY-R mice. Chemogenetic activation of these neurons promoted resilience to STA, while their inhibition increased susceptibility to STA. The study also identified two distinct downstream circuits of VTA glutamatergic neurons: the VTA to nucleus accumbens (NAc) shell projection, which regulates the development of resilience, and the VTA to lateral habenula (LHb) projection, which maintains the established resilient phenotype.

In conclusion, the studies by Ai et al.^6^ and Han et al.^7^ represent major breakthroughs in the field of pain research. By establishing a new paradigm for studying individual differences in pain susceptibility and resilience, they have provided valuable insights into the neural mechanisms underlying these differences. The identification of DRN GABAergic and VTA glutamatergic neurons as key regulators of pain susceptibility and resilience opens promising avenues for future research and the potential to revolutionize our understanding of pain and its treatment.^9^ More broadly, after screening out the susceptible subgroup from BY mice, future research can focus exclusively on these mice to uncover more precise pathological targets for pain sensation.^9^, ^10^ Simultaneously, by studying the brain mechanisms of those resilient to pain sensation, researchers could identify new drug targets that enhance the natural ability to cope with pain.^9^, ^10^ These drug targets could facilitate the development of safer and more effective pain medications, thereby improving the quality of life for millions of individuals suffering from pain conditions. This undoubtedly opens up a conceptually novel research direction and a new strategy for clinical analgesia.

While these studies represent significant advancements, several questions remain to be addressed. First, the findings open the door for the development of novel analgesic drugs targeting DRN GABAergic, as well as VTA glutamatergic neurons and its downstream circuits. Future research could focus on identifying additional molecular targets within these neurons and testing the efficacy of pharmacological interventions in both animal models and human patients.

Second, in the study by Han et al., the researchers identified a series of brain regions in which the expression of c-Fos protein changed after mice experienced the modeling paradigm.^7^ This suggests that these brain regions may also serve as important targets for regulating STA susceptibility or resilience. Investigating the cellular, circuit, and molecular mechanisms through which these brain regions function represents a significant direction for future research. Additionally, exploring the connections between these brain regions and the VTA and/or DRN holds considerable potential as a promising research avenue. Interestingly, the expression of c-Fos protein in some brain regions either decreased (e.g., in DRN serotonergic neurons ^6^) or increased (e.g., in paraventricular thalamic nucleus neurons^7^) in both susceptible and non-susceptible animals. This suggests that all BY mice may exhibit other comorbid symptoms, such as anxiety-like behaviors.^6^ This represents a highly intriguing direction for future research, as this phenomenon has often been overlooked in previous studies.

Third, the genetic and epigenetic factors contributing to individual differences in pain susceptibility and resilience are still poorly understood. To address this gap, future research could explore the role of specific genes and epigenetic modifications in shaping STA susceptibility and resilience.

Another promising direction is the exploration of social hierarchy and its impact on pain susceptibility and resilience. The studies briefly mention the potential role of social rank in modulating pain responses, but this aspect remains underexplored. Future research could incorporate behavioral tests, such as the tube test, to assess how social dominance influences the development of pain susceptibility and resilience.

Finally, although both Ai et al. and Han et al. primarily focused on male mice, their behavioral data indicated that female C57BL/6 J mice also displayed distinct susceptible and resilient phenotypes following STA exposure, in proportions comparable to those in males.^7^ However, a key limitation of these studies is that they did not examine the mechanisms underlying susceptibility or resilience to STA in female mice, leaving this an important and underexplored question. These two studies identified DRN GABAergic neurons and VTA glutamatergic circuits as mediators of susceptibility or resilience to STA. However, whether these mechanisms underlie the inter-individual differences in responses under other pain conditions—such as visceral pain disorders induced by early-life stress^12^—remains unknown. This represents an important question for future investigation.

## CRediT authorship contribution statement

**Hongxing Zhang:** Writing – review & editing, Writing – original draft, Supervision, Project administration, Conceptualization. **Xin Yang:** Funding acquisition. **Lin Ai:** Writing – review & editing, Writing – original draft, Conceptualization. **Yi Han:** Writing – review & editing, Writing – original draft, Methodology, Conceptualization. All authors have read and agreed to the published version of the manuscript.

## Consent for publication

Not applicable.

## Ethical statement

Not applicable.

## Funding

The present study was supported by 10.13039/501100001809National Natural Science Foundation of China (31970937, 82271255), Jiangsu Province Key R&D Program Social Development Project (BE2023690) and the Postgraduate Research and Practice Innovation Program of Jiangsu Province (KYCX22_2918_Han, KYCX23_2952_Ai, and KYCX24_3105_Yang).

## Declaration of competing interest

The authors declare that they have no known competing financial interests or personal relationships that could have appeared to influence the work reported in this paper. Hongxing Zhang is an Editorial Board Member for this journal and was not involved in the editorial review or the decision to publish this article.
